# Analysis of glomerular PLA2R efficacy in evaluating the prognosis of idiopathic membranous nephropathy in the background of different serum anti-PLA2R levels

**DOI:** 10.1080/0886022X.2022.2068442

**Published:** 2022-04-28

**Authors:** Yuemeng Sun, Ping Lan, Jie Feng, Zhigang Wang, Chao Liu, Liyi Xie, Xiaoyang Yu

**Affiliations:** Department of Nephrology, Kidney Hospital, The First Affiliated Hospital of Xi’an Jiaotong University, Xi’an, China

**Keywords:** Membranous nephropathy, glomerular PLA2R, serum anti-PLA2R, clinical remission

## Abstract

**Objective:**

To verify glomerular PLA2R antigen and serum PLA2R antibody expression in membranous nephropathy as well as to explore glomerular PLA2R efficacy in evaluating the prognosis of idiopathic membranous nephropathy (IMN) in the background of different serum anti-PLA2R levels.

**Methods:**

We retrospectively analyzed 155 patients who were diagnosed with IMN by kidney biopsy. Patients were divided into six groups according to their serum PLA2R antibody or glomerular PLA2R antigen positiveness and the level of serum anti-PLA2R titer. Both clinical features and pathological characteristics were recorded, and the remission time was compared among groups. Correlation between clinical figures and the anti-PLA2R titer or semi-quantity of glomerular PLA2R antigen was detected.

**Results:**

A positive correlation between time to partial remission and serum anti-PLA2R titer was found. Among patients with serum anti-PLA2R titer <150 RU/ml, there were shorter remission time in negative glomerular PLA2R antigen group compared with positive glomerular PLA2R antigen, and a positive correlation between time to complete remission and semi-quantity of glomerular PLA2R antigen was found.

**Conclusion:**

Both glomerular PLA2R antigen and serum anti-PLA2R play a role in disease presentation and prognosis in primary membranous nephropathy. Glomerular PLA2R antigen has a major role on disease prognosis when serum anti-PLA2R titer is less than 150RU/ml, while serum anti-PLA2R has predominant role in IMN prognosis when serum anti-PLA2R titer is above 150RU/ml.

## 1. Introduction

Membranous nephropathy (MN) is characterized by the deposition of immune complexes in the subepithelial space and the diffuse thickening of the glomerular basement membrane, which is now the leading cause of nephrotic syndrome in adults [1]. The M-type phospholipase A2 receptor (PLA2R), first discovered by Beck, which located on cell surface of podocytes, is the major auto-antigen in most patients with idiopathic membranous nephropathy (IMN) [2]. Previous studies mainly focused on the serum antibody status of patients with IMN. It is generally believed that the levels of antibody titer are correlated with disease severity and prognosis [3–5], in which higher titer predicted more severe proteinuria and less chance to remission. However, there are few studies on glomerular PLA2R antigen in evaluating the prognosis of the disease, especially the relationship between different levels of glomerular PLA2R antigen combined with serum PLA2R antibody and disease risk. Based on the stratification of serum PLA2R antibody levels, our study analyzed the different effects of glomerular PLA2R antigen on the prognosis assessment of IMN, and further explained how to evaluate the disease risk by combining glomerular PLA2R antigen and serum PLA2R antibody.

## 2. Methods

### 2.1. Patients

**Figure 1.**
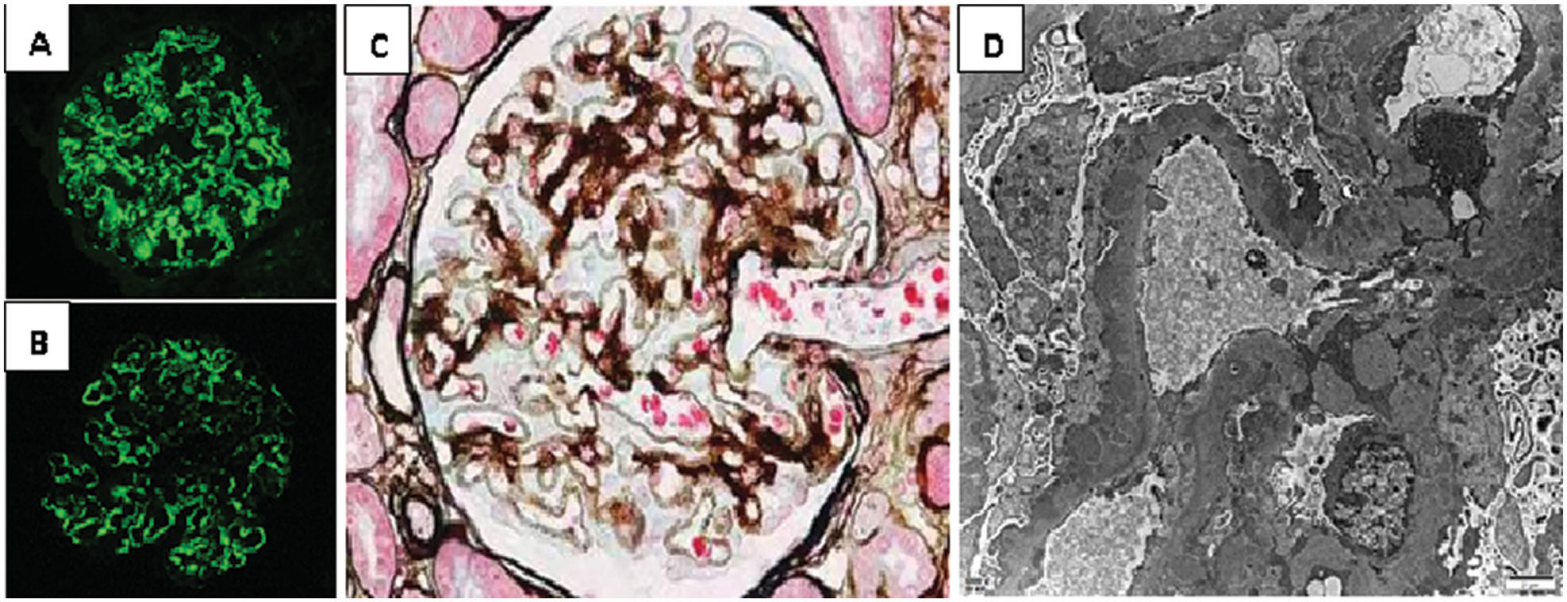
Renal pathological manifestations of biopsy-proven idiopathic membranous nephropathy. (A) IgG4 immunofluorescence in frozen section of renal biopsy. (B) The immunofluorescence of renal tissue PLA2R in paraffin section. Granular immune complex deposition along glomerular capillary wall can be observed in both of (A) and (B). (C) Light microscopy of membranous nephropathy (PASM staining, 400×). It displays glomerular basement membrane thickening and spike formation with the silver stain. (D) Electron microscopy of membranous nephropathy (6000×). A large amount of electron dense deposits are found in sub-epithelial area. It shows diffuse spike formation and foot process effacement.

**Figure 2.**
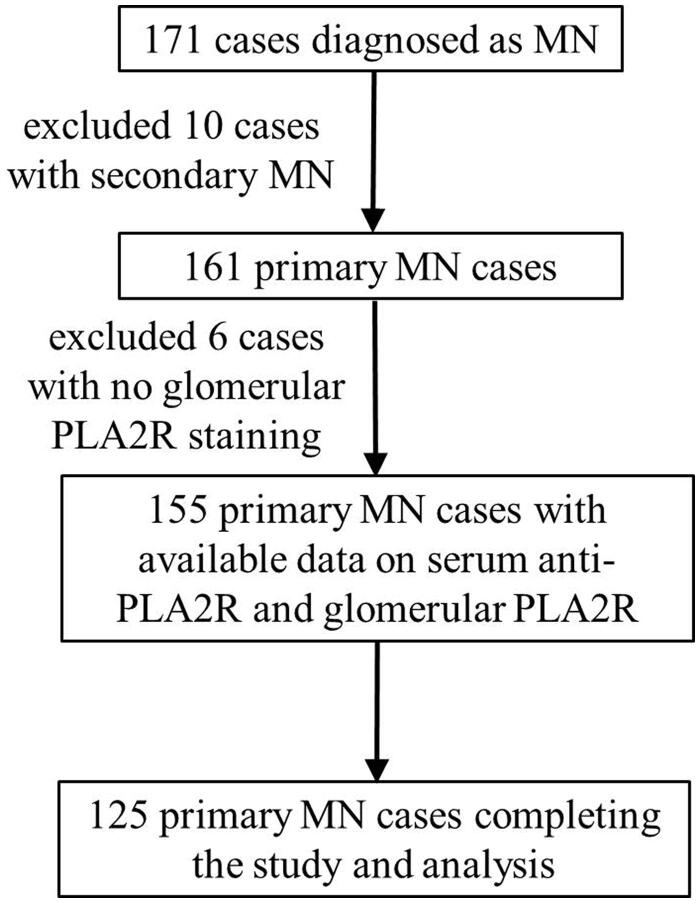
The flow-chart of cases included in each step. One hundred and seventy-one cases were initially eligible, 10 cases of secondary MN, and 6 cases without glomerular PLA2R staining were excluded, and 125 cases were followed up and completed the study and analysis.

It was a retrospective cohort study. A total of 155 patients with biopsy-proven MN collected between June 2018 and June 2019 were examined for serum PLA2R antibody and glomerular PLA2R antigen as baseline data at the same time of biopsy (Figure 1). 125 cases were followed up and completed the study and analysis (Figure 2). Patients with secondary MN, including autoimmune diseases (lupus nephritis, rheumatoid arthritis, etc.), infection-related MN (HBV-MN), and MN with malignancies or exposure to toxic agents, were excluded. Patients were divided into six groups according to their serum PLA2R antibody or glomerular PLA2R antigen positiveness and the level of serum PLA2R antibody titer. Of the included individuals, baseline clinical features at kidney biopsy were compared among six groups, including urinary protein excretion, serum albumin level. Immunosuppressive therapy was commonly started in moderate- or high-risk patients with severe nephrotic syndrome, proteinuria not responding to supportive therapy or deteriorated renal function. The patients of receiving immunosuppressive therapy included in this study were all treated with calcineurin inhibitor (CNI) combined with glucocorticoid, with or without angiotensin receptor blocker (ARB), so as to avoid the influence of different treatment regimens on the remission time of IMN. Remission rate and remission time after the start of immunosuppressive therapy was also compared in patients with data available. Time to remission was recorded according to the following criteria: Complete remission (CR) was defined as urinary protein excretion <0.5 g/day (urine protein-to-creatinine ratio [Upcr] < 500 mg/g); partial remission (PR) was defined as a 50% or greater reduction from peak values of urinary protein and urinary protein excretion <3.5 g/day (Upcr <3500 mg/g).

### 2.2. Measurement of serum anti-PLA2R, detection of PLA2R antigen, and IgG subclasses in renal biopsy

Circulating PLA2R antibodies were measured in 155 patients with IMN simultaneously at the time of biopsy. Serum samples were measured by anti-PLA2R ELISA kit (EUROIMMUN AG, Lübeck, Germany). The results were considered as negative for <20 RU/ml and positive for ≥20 RU/ml [6]. Renal biopsy specimens were divided and processed for light, immunofluorescence (IF) and electron microscopy (EM) analysis. To detect presence of PLA2R antigen, sections of biopsied tissue were de-paraffinized, hydrated, and heated for 10 min at 120 °C before being blocked with 10% FBS for 10 min. The antigens were then conjugated using a rabbit polyclonal anti-human PLA2R antibody (Atlas Antibodies) followed by an FITC-conjugated swine anti-rabbit IgG antibody (Dako, Tokyo, Japan) [7]. Renal biopsy specimens were analyzed by three pathologists independently and the final fluorescence intensity value was determined by the average value of the results. Semi-quantity of PLA2R antigen and IgG subclass was calculated by the fluorescence intensity on glomerular staining as follows: negative, 0; very weak, 0.5; weak, 1; moderate, 2; strong, 3.

### 2.3. Statistical analysis

The statistical analyses were conducted using SPSS version 19.0 (SPSS, Chicago, IL). As for the data description, continuous variables with symmetric distribution were presented as mean ± standard deviation (SD), while non-symmetrically distributed variables as medians (25–75% interquartile range). The *t*-test was used for parametric analysis and the Mann–Whitney *U*-test was used for nonparametric analysis. Categorical variables were described as frequencies or percentages, and the data were analyzed with Pearson’s Chi-square test or Fisher’s exact test. The bivariate correlation was analyzed by Pearson correlation analysis, and *R*^2^ was the coefficient of determination. *p* values <0.05 were considered as statistically significant.

#### 2.3.1. Study approval

All methods were carried out in accordance with the guidelines and regulations of National Health and Family Planning Commission. All the experimental protocols were approved by the Ethics Committee of Xi'an Jiaotong University, China. And we confirm that informed consent was obtained from all subjects, if subjects were under 18, from a parent and/or legal guardian.

## 3. Results

One hundred and fifty-five cases of primary MN were enrolled in the study (Figure 2) and baseline characters are listed in Table 1.

**Table 1.** Baseline characteristics of IMN patients.

		Range
Age (years)	54.1 ± 11.8	25–86
Gender (M/F)	117/38	
BMI (kg/m^2^)	21.3 ± 3.7	17.1–29.9
Systolic Bp (mmHg)	133 ± 25.6	102–164
Diastolic Bp (mmHg)	75 ± 15.3	52–98
Serum albumin (g/L)	23.1 ± 6.5	8.8–40.0
Urinary protein (g/24h)	4.4 ± 2.1	0.7–10.5
Duration of proteinuria (months)	5.0 ± 3.5	1.0–13.0
eGFR (ml/min/1.73m^2^)	89.6 ± 12.8	54–125
Serum creatinine (µmol/L)	72.5 ± 9.4	47–98
Cholesterol (mmol/L)	5.89 ± 2.42	2.82–8.96
Triglycerides (mmol/L)	1.89 ± 0.56	0.42–3.36
Treatment (*n*, %)		
ARB	37/155 (23.9%)	
GC + CNI	118/155 (76.1%)	
Follow-up time (months)	12 (7.5, 16.5)	6–23

BMI: body mass index; Bp: blood pressure; GFR: glomerular filtration rate; ARB: angiotensin receptor blocker; GC: glucocorticoid; CNI: calcineurin inhibitor.

### 3.1. Glomerular PLA2R antigen expression in idiopathic membranous nephropathy

**Figure 3.**
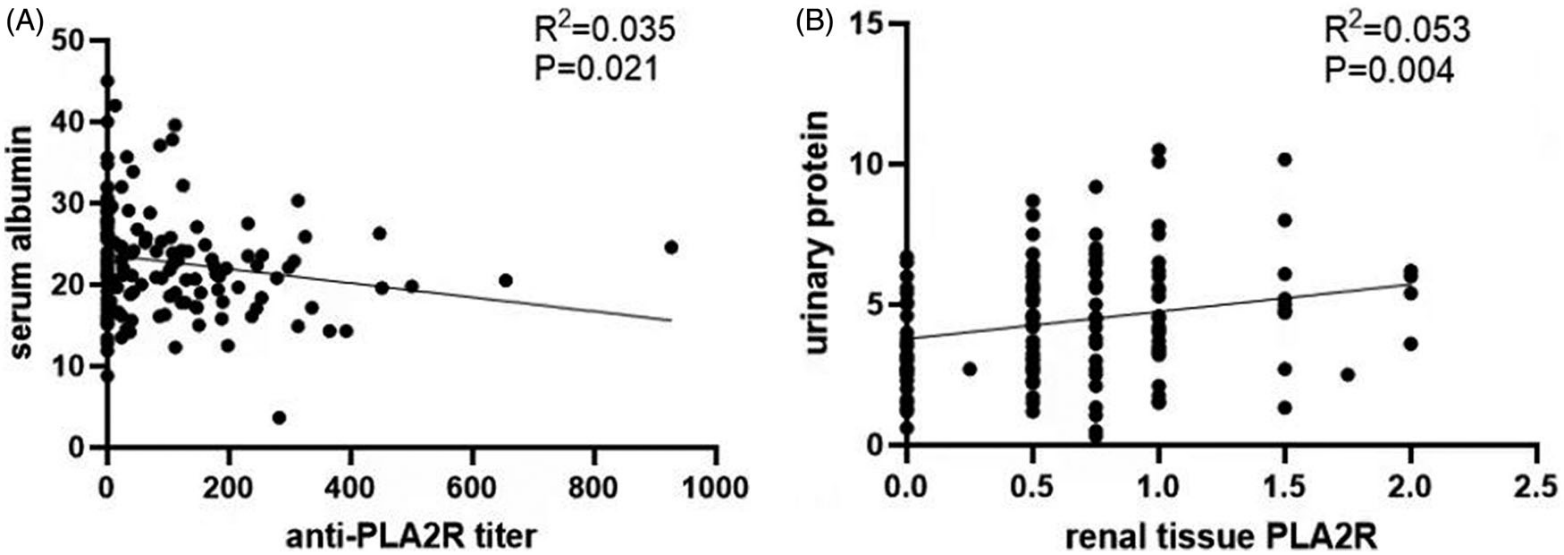
Correlation between serum anti-PLA2R antibody or renal tissue PLA2R and basic clinical indicators. (A) Correlation of serum anti-PLA2R titer and serum albumin. It shows a slightly negative correlation between serum albumin and serum anti-PLA2R titer (*R*= −0.19, *p* = 0.021). (B) Correlation of renal tissue PLA2R antigen and quantity of proteinuria. It displays a positive correlation between urinary protein and the intensity of PLA2R antigen expression in renal tissue (*R* = 0.23, *p* = 0.004). Semi-quantity of PLA2R antigen was calculated by the fluorescence intensity on glomerular staining as follows: negative, 0; very weak, 0.5; weak, 1; moderate, 2; strong, 3.

Clinical differences between serum anti-PLA2R positive and negative patients with IMN are shown in Table 2. There were higher PLA2R antigen (*p* = 0.048) and IgG4 (*p* = 0.057) expression in glomeruler tissue in the serum anti-PLA2R positive group compared with the negative group. It showed a slightly negative correlation between serum albumin and serum anti-PLA2R titer (*R*= −0.19 *p* = 0.021) (Figure 3(A)). However, serum anti-PLA2R titer was not correlated to PLA2R antigen expression in tissue.

**Table 2.** Clinical presentation and prognosis of groups regarding to different serum anti-PLA2R titer.

	Serum anti-PLA2R ≥ 150	Serum anti-PLA2R < 150	Serum anti-PLA2R negative
	(*n* = 35)	(*n* = 61)	(*n* = 59)
Serum albumin	20.5 ± 5.0*	22.2 ± 6.6*	23.7 ± 7.1
Urinary protein	5.1 ± 1.9	4.0 ± 2.2	4.2 ± 2.0
Percentage of nephrotic syndrome	28/35 (80.0%)	36/61 (59.0%)	31/59 (52.5%)
Glomerular PLA2R positive	31/35	38/61	45/59
Glomerular PLA2R semi-quantity	0.69 ± 0.45	0.69 ± 0.45	0.54 ± 0.55
NR	35/78 (44.9%)		16/47 (34.0%)
PR/CR	43/78 (55.1%)		31/47 (66.0%)
CR	20/78 (25.6%)		20/47 (42.6%)

*p < 0.05, anti-PLA2R ≥150 RU/ml versus anti-PLA2R <150 RU/ml.

CR: complete remission; PR: partial remission; NR: no response.

The immunofluorescence of glomerular PLA2R and IgG4 of IMN are shown in Figure 1. The urinary protein levels tended to be lower in glomerular PLA2R antigen negative IMN patients than in positive patients (*p* = 0.017), while there was no significant difference with respect to serum albumin between the two groups. Histopathologically, there was higher expression intensity of IgG4 along with PLA2R antigen in glomerular capillary in positive PLA2R group than the negative group (*p* = 0.007).

It showed a positive correlation between urinary protein and the intensity of PLA2R antigen expression in renal tissue (*R* = 0.23, *p* = 0.004) (Figure 3(B)). What’s more, renal IgG4 expression was positive correlated to PLA2R intensity in renal tissue, (*r* = 0.22, *p* = 0.006). The remission rate was not different between two groups (59.7% versus 58.7%, *p* > 0.05) (Table 3).

**Table 3.** Clinical presentation and prognosis of groups regarding to different glomerular PLA2R antigen intensity.

	Glomerular PLA2R positive	Glomerular PLA2R negative
	(*n* = 114)	(*n* = 41)
Serum albumin	22.2 ± 6.1	23.4 ± 7.9
Urinary protein	4.8 ± 2.1*	3.6 ± 1.7*
Serum anti-PLA2R positive	69/114 (60.5%)	27/41 (65.9%)
Serum anti-PLA2R titer	99.3 ± 118.9	86.9 ± 186.6
Renal IgG4 semi-quantity	1.7 ± 0.57*	1.6 ± 0.76*
NR	35/92 (38.0%)	15/33 (45.5%)
PR/CR	57/92 (62.0%)	18/33 (54.5%)
CR	33/92 (35.9%)	9/33 (27.3%)

*p < 0.05, glomerular PLA2R positive versus glomerular PLA2R negative.

CR: complete remission; PR: partial remission; NR: no response.

### 3.2. Combination of glomerular PLA2R antigen and serum PLA2R antibody to evaluate clinical outcome in primary MN

We detected positive serum PLA2R antibody or glomerular PLA2R antigen in 77.42% patients with IMN. Based on the stratification of serum PLA2R antibody levels, our study analyzed the different effects of glomerular PLA2R antigen on the prognosis assessment of IMN.

#### 3.2.1. Comparison of remission rates among groups with different baseline levels of serum PLA2R antibody and glomerular PLA2R

Patients were divided into six groups according to their serum PLA2R antibody or glomerular PLA2R antigen positiveness. And the patients of serum anti-PLA2R positive were further divided into high titer and low titer group defined by anti-PLA2R titer above or below 150 RU/ml, according to the distribution characteristics of the serum anti-PLA2R range [median about 150 RU/ml (42.36, 241.25)], so as to detect the relationship between serum PLA2R antibody or glomerular PLA2R antigen and clinical remission rate. Patients with both negative serum PLA2R antibody and glomerular PLA2R antigen presented with higher complete remission rate compared with other groups (*p* < 0.05). Among patients with positive glomerular PLA2R antigen, there was higher remission rate in group of lower serum PLA2R antibody titer (<150 RU/ml) compared with higher titer group (≥150 RU/ml). Results showed that patients who were non-PLA2R-related MN experienced a higher complete remission rate than PLA2R related patients (*p* < 0.001) (Table 4).

**Table 4.** Remission rate of different groups evaluated by combination of serum anti-PLA2R and glomerular PLA2R antigen.

	Remission rate (*n*, %)					
	Serum^−^ & tissue^-^	Serum^−^ & tissue^+^	Serum < 150 & tissue^−^	Serum < 150 & tissue^+^	Serum ≥ 150 & tissue^−^	Serum ≥ 150 & tissue^+^
CR*	5/14	9/33	5/19	9/31	2/4	6/24
	35.7%	27.3%	26.3%	29.0%	50.0%	25.0%
PR + CR	8/14	24/33	13/19	15/31	4/4	14/24
	57.1%	72.7%	68.4%	48.4%	100%	58.3%
NR	6/14	9/33	6/19	16/31	0/4	10/24
	42.9%	27.3%	31.6%	51.6%	0	41.7%

*p < 0.001, Complete remission rates were different among six groups.

CR: complete remission; PR: partial remission; NR: no response.

#### 3.2.2. Comparison of the effects of glomerular PLA2R on remission time in the background of different serum anti-PLA2R levels

Among 40 patients achieved complete remission, there was shorter time to complete remission in patients with negative tissue PLA2R antigen and positive serum PLA2R antibody but less than 150 RU/ml compared with PLA2R antibody above 150 RU/ml (*p* = 0.032). There was shorter remission time in the group of serum PLA2R antibody <150 RU/ml and negative PLA2R antigen in renal tissue compared with the group of negative serum PLA2R antibody and positive tissue PLA2R antigen (*p* = 0.014) (Table 5). However, no differences were found in partial remission time.

**Table 5.** Time to remission of different groups evaluated by combination of serum anti-PLA2R and glomerular PLA2R antigen.

	Remission time (months)					
	Serum^−^ & tissue^-^	Serum^−^ & tissue^+^	Serum < 150 & tissue^−^	Serum < 150 & tissue^+^	Serum ≥ 150 & tissue^−^	Serum ≥ 150 & tissue^+^
PR	3.0 (1.75–5.5)	4.0 (2.0–5.5)	4.0 (2.25–6.0)	3.0 (3.0–6.5)	6.0 (4.0–8.0)	8.0 (3.0–9.0)
CR	6.0 (3.0–8.5)	8.0 (6.0–11.0)*	4.0 (2.0–6.0)*^a#^	5.5 (3.25–9.0)	9.0 (7.0–11.0)^a^	10.5 (3.25–11.75)^#^

Time to complete remission: *serum < 150 & tissue^−^ versus serum^−^ & tissue^+^, *p* = 0.014.

^a^Serum < 150 & tissue^−^ versus serum ≥ 150 & tissue^−^, *p* = 0.032.

^#^Serum < 150 & tissue^−^ versus serum ≥ 150 & tissue^+^, *p =* 0.036.

CR: complete remission; PR: partial remission.

### 3.3. Correlation between time to remission and serum PLA2R antibody or glomerular PLA2R antigen

**Figure 4.**
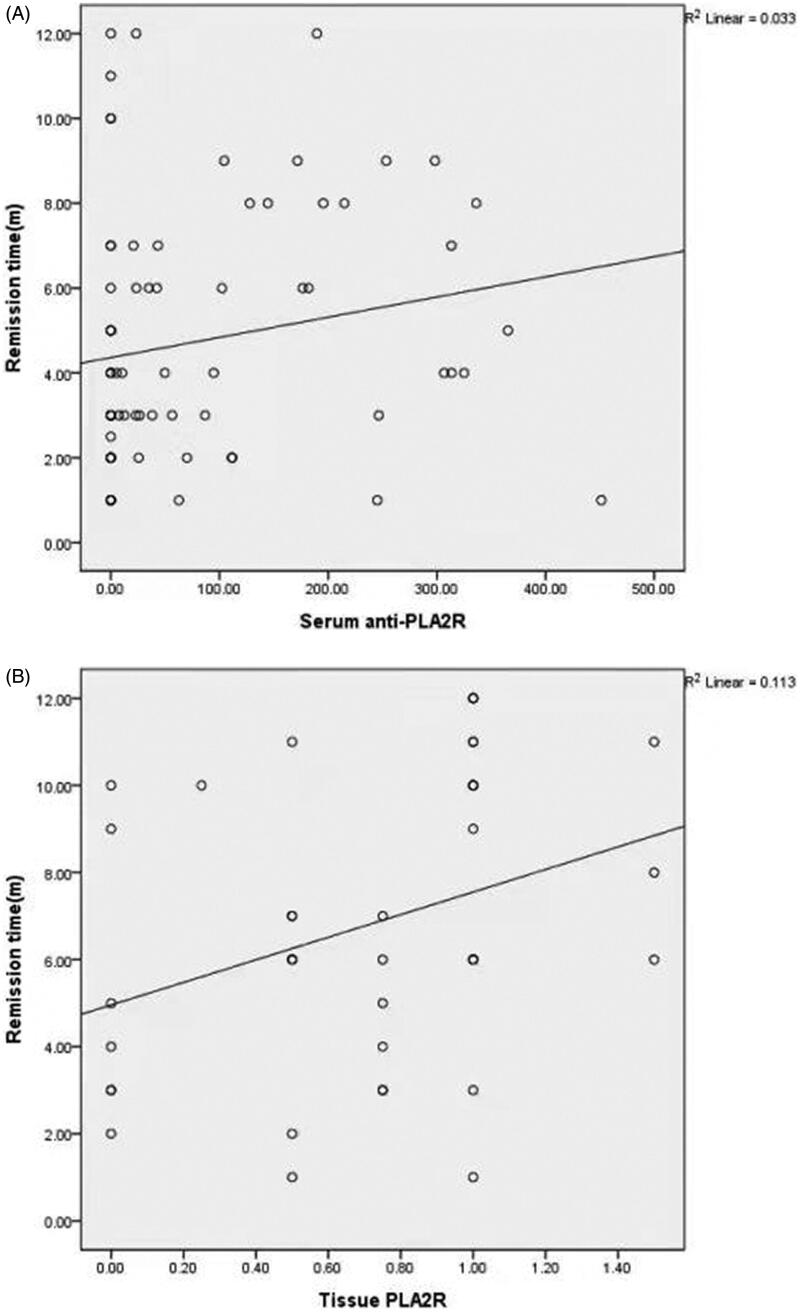
Correlation between time to remission and serum anti-PLA2R titer or tissue PLA2R. (A) Correlation between time to partial remission and serum anti-PLA2R titer. It exhibits a positive correlation between time to partial remission and serum anti-PLA2R titer (*R* = 0.25, *p* = 0.03). (B) Correlation between time to complete remission and semi-quantity of tissue PLA2R antigen. It shows a positive correlation between time to complete remission and renal tissue PLA2R antigen (*R* = 0.385, *p* = 0.01). Semi-quantity of PLA2R antigen was calculated by the fluorescence intensity on glomerular staining as follows: negative, 0; very weak, 0.5; weak, 1; moderate, 2; strong, 3.

In order to explore the role of PLA2R antibody in prediction of MN prognosis, we further detect whether there is a correlation between serum anti-PLA2R titer or renal tissue intensity and time to remission. Among 74 patients achieved partial remission in proteinuria, a positive correlation was found between time to partial remission and serum anti-PLA2R titer (*R* = 0.25, *p* = 0.03) (Figure 4(A)). Among 40 patients achieved complete remission in proteinuria, a positive correlation was found between time to complete remission and semi-quantity of tissue PLA2R antigen. (*R* = 0.385, *p* = 0.01) (Figure 4(B)).

**Figure 5.**
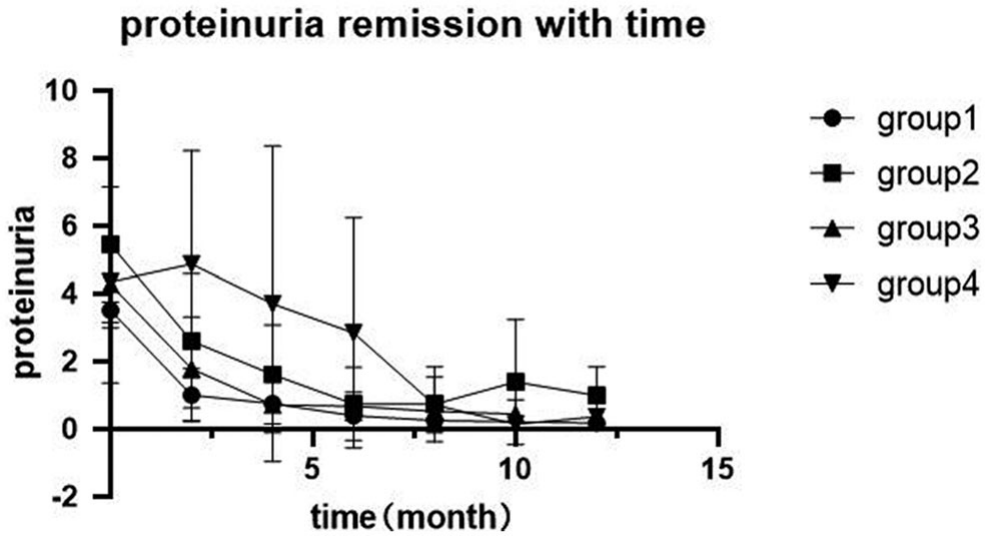
Changes of proteinuria with time in each group. Group 1: serum < 150 RU/ml & tissue^−^; Group 2: serum < 150 RU/ml & tissue^+^; Group 3: serum ≥ 150 RU/ml & tissue^−^; Group 4: serum ≥ 150 RU/ml & tissue^+^.

**Figure 6.**
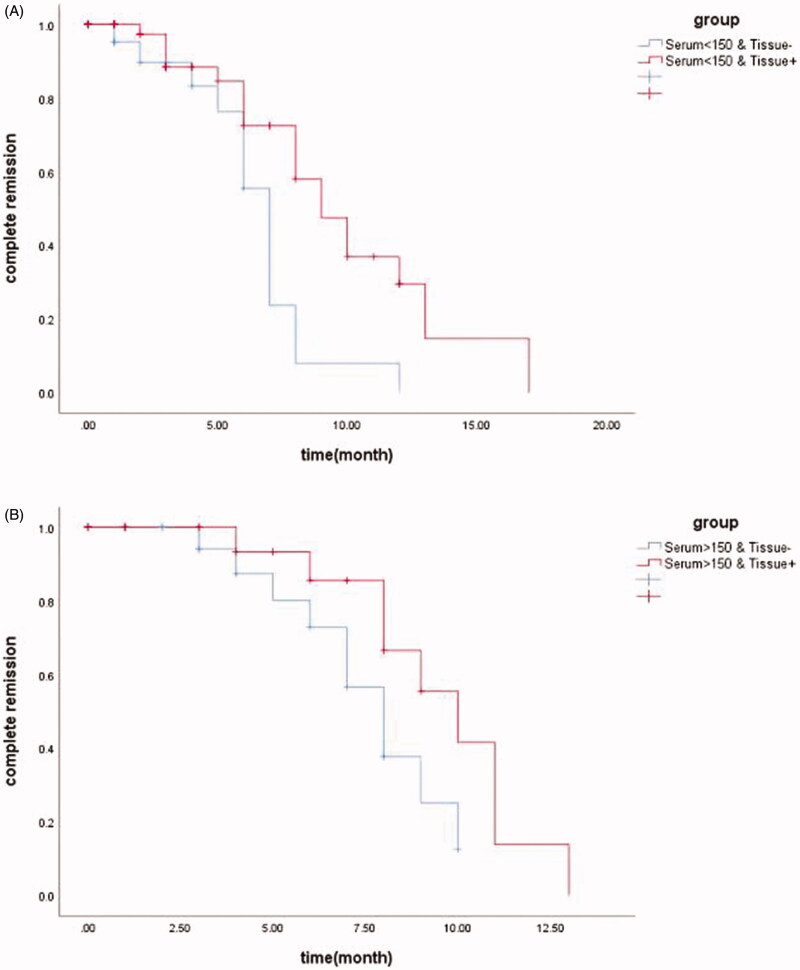
Kaplan–Meier analysis of renal PLA2R for IMN remission time evaluation in the background of different serum anti-PLA2R levels. (A) Kaplan–Meier analysis showed shorter remission time in group serum < 150 RU/ml & tissue^−^ compared to serum < 150 RU/ml & tissue^+^ (median remission time 7 month versus 9 month, *p* = 0.004). (B) Kaplan–Meier analysis showed no significant difference in remission time between group serum > 150 RU/ml & tissue^−^ compared to serum > 150 RU/ml & tissue^+^ (median remission time 8 month versus 10 month, *p* = 0.082).

Regarding to 39 patients in complete remission, the decline in urinary protein and remission rate varies in each group with different serum PLA2R antibody or glomerular PLA2R antigen (Figure 5). Among patients with positive serum anti-PLA2R antibody and below 150 RU/ml, 13 patients achieved complete remission over 12 months follow-up. By Kaplan–Meier analysis, we observed shorter remission timer in serum < 150R U/ml & tissue^−^ group compared to serum < 150 RU/ml & tissue^+^ group, with the median remission time being 7 month and 9 month, respectively (*p* = 0.004) (Figure 6(A)). On the other hand, there was no significant difference in remission time between patients with serum > 150 RU/ml & tissue^−^ and serum > 150 RU/ml & tissue^+^ by Kaplan–Meier analysis (*p* = 0.082) (Figure 6(B)).

## 4. Discussion

It has been reported about the value of either serum PLA2R antibody or glomerular PLA2R antigen to diagnosis of primary MN with 50–80% sensitive and almost 100% specific [8, 9], now our study displays 75.3% patients with IMN had serum PLA2R antibody or tissue PLA2R antigen and no PLA2R positivity in secondary MN or other primary glomerulus. As regard to patients with no expression in serum or renal tissue, we assume that other antigens are responsible for disease development such as thrombospondin type-1 domain-containing 7 A (THSD7A) [10] or recently recognized Exostosin 1 and Exostosin 2 [11]. Among patients diagnosed with PLA2R related membranous nephropathy, we also observed the discrepancy of the positivity between serum anti-PLA2R and glomerular PLA2R, with 23/97 cases presented positive serum anti-PLA2R and negative PLA2R staining in renal tissue, while 14/97 cases presented the opposite results. A possible explanation to this divergence is the timing of biopsy in relation to the disease course [12, 15]. In the former scenario, serum anti-PLA2R may be negative because of the early stage in the disease course, on the other hand, the patients may already achieved immunological remission. The latter scenario is less common, which has been reported earlier [13,14] and the possible explanations may be that these antibodies were not pathogenic or that some specific epitope of PLA2R antigen were poorly detectable in kidney biopsy [15].

While some studies found an association between the degree of proteinuria or serum albumin and serum anti-PLA2R titer at a defined time point, which is consistent to our results [16–20], others found only a weak or no association [21–23]. Such variability likely reflects the time lag between immunologic and clinical activity, and indeed, a latency period as long as 8 months has been observed between the presence of serum anti-PLA2R and the first clinical manifestations of MN.

Several studies demonstrated that strong immunofluorescence staining of glomerular IgG2 is frequently seen with cancer-associated MN, whereas the dominance of deposits of IgG4 generally favors IMN [24, 25]. In primary MN, the dominant IgG subclass of PLA2R antibody is IgG4 [3,26,27], which can be observed co-deposition of PLA2R and IgG4 in renal tissue. Consistent with that conclusion, we observed 49 cases with both deposition of PLA2R and IgG4 in our study. However, the remaining 43 cases showed predominant IgG4 but no PLA2R, which may indicate some other antigen can also bind to IgG4 antibody such as THSD7A [28]. As for cases showed PLA2R but no predominant IgG4 deposition, Hofstra’s study [6] revealed that the anti-PLA2R antibody was not confined to IgG4 subclass in 5–7% cases which may give explanation to this scenario.

Lots of studies had confirmed that low baseline serum anti-PLA2R levels predict subsequent spontaneous remission [6, 29], whereas high baseline anti-PLA2R levels are associated with development of nephrotic syndrome in patients with initial non-nephrotic proteinuria and with progressive loss of kidney function [30–32]. Consistent to that conclusion, we observed higher complete remission rate in serum anti-PLA2R titer <150 RU/ml among PLA2R-associated MN. What’s innovative of this research is that we observed shorter remission time in serum anti-PLA2R <150 RU/ml and negative PLA2R antigen in renal tissue. Our results indicated that glomerular PLA2R had a major role on disease prognosis when serum anti-PLA2R titer was less than 150 RU/ml, which means with PLA2R antigen intensity in renal tissue increased, the time to clinical remission became longer. However, when serum anti-PLA2R titer was high (≥150 RU/ml), serum anti-PLA2R played a predominant role in the disease remission. As serum anti-PLA2R titer increased, the time to clinical remission became longer.

PLA2R antigen in kidney tissue can be detected in a large proportion of patients who were negative for serum anti-PLA2R and provide a more reliable means by which to diagnose IMN [33–35], though there was no agreement on the role of glomerular PLA2R antigen in prognosis of MN. One research [36] found higher remission rate after immunosuppressant therapy in the PLA2R antigen positive group. On the contrary, another Chinese cohort [37] found higher remission rates in non-PLA2R-associated MN compared with PLA2R-associated MN at both 3rd month and 6th month landmark analysis. We found no differences in remission rate between baseline positive and negative glomerular PLA2R antigen group. However, as mentioned above, among patients with serum anti-PLA2R titer <150 RU/ml, our results revealed shorter remission time in the negative glomerular PLA2R group compared with the positive glomerular PLA2R group, which suggests that negative glomerular PLA2R antigen with low titer of serum antibody may be associated with a better response to therapy.

In general, patients with high serum anti-PLA2R titers have a higher proportion of PLA2R positivity in renal tissue. Our study showed that the positive rate of glomerular PLA2R with serum anti-PLA2R titer above 150 RU/ml was 88.6%, which was a little lower than previous studies [38]. One possible explanation of the low positiveness of glomerular PLA2R antigen may be due to the fact that epitopes are poorly accessible at the time of kidney biopsy or reflecting a technical artifact [39]. Luo [40] found that patients with high level of serum anti-PLA2R but negative PLA2R antigen by immunofluorescence on frozen tissue (IF-F) method proved positive for glomerular PLA2R antigen by immunohistochemistry method. It could be explained that epitopes were easily occupied by a large number of antibodies, so we could not detect the target antigen by IF-F until an antigen retrieval step was added to expose epitopes and reveal the presence of PLA2R antigen using the immunohistochemistry procedure. In the future, further research needs to double detection of glomerular PLA2R by IF-F and immunohistochemistry to further explore the effect of glomerular PLA2R on the prognosis of IMN.

In order to avoid the influence of different treatment regimens on the remission time of IMN, the patients of receiving immunosuppressive therapy included in this study were all treated with calcineurin inhibitor combined with glucocorticoid, with or without ARB. Therefore, this study excluded the confounding factor of initial treatment regimens. What's more, apart from PLA2R antibody or antigen, sustained hypertension, impaired renal function, and significant chronic tubulointerstitial lesions in the initial renal biopsy all exert influences on remission rate in MN patients, and also portend an unfavorable outcome [41]. Li et al. [42] revealed lower remission rate in IMN patients with FSGS lesion and chronic tubular lesions when treated with immunosuppresive agents. In the future, large sample, prospective cohort studies will be carried out to analyze the correlation between the therapy and remission time and the possible role of chronic lesions in reducing the response rate in a stratified manner.

Only a few research explored the relationship between the PLA2R antigen in renal tissue and MN prognosis. A Chinese center [43] included MN patients with repeated biopsy found that patients with increasing or stable expression of glomerular PLA2R in second biopsy tended to achieve no remission or disease relapse. The results do not support the uselessness of the kidney biopsy in the presence of anti-PLA2R antibodies and do not allow to support the use of the anti-PLA2R titer alone to define the treatment. Our research underlines a persistent role of histological investigation.

Our study demonstrates the role of glomerular PLA2R antigen and serum PLA2R antibody in the combined evaluation of the prognosis in primary MN. In conclusion, based on the stratification of serum PLA2R antibody levels, this study analyzed the different effects of glomerular PLA2R antigen on the prognosis assessment of IMN, and further explained how to evaluate the disease risk by combining serum anti-PLA2R and glomerular PLA2R antigen. Certainly, our research still exist some limitations. First, it is only a single-center clinical research with limited cases, so selection bias could not be avoided. Second, we did not detect serial serum anti-PLA2R titer and had not conducted repeated kidney biopsy, so we could not dynamically monitor the tendency of anti-PLA2R titer and PLA2R antigen expression. At last, since it is a retrospective study, a subgroup analysis was performed based on the distribution characteristics of the serum anti-PLA2R range [median about 150 RU/ml (42.36, 241.25)], and prospective studies need to be completed in the future to analyze the anti-PLA2R cutoff point that may affect glomerular PLA2R to evaluate the prognosis of IMN, and analyze the combined effect of the two on IMN prognosis. Further study of basic researches at the molecular level are needed to confirm the pathological role of PLA2R antigen in kidney tissue, so as to provide accurate judgment to the prognosis of the disease as well as more effective and individualized therapy for patients with primary membranous nephropathy.

## Data Availability

All data generated or analyzed during this study are included in this article and its supplementary information files.
